# Influenza, dengue and common cold detection using LSTM with fully connected neural network and keywords selection

**DOI:** 10.1186/s13040-022-00288-9

**Published:** 2022-02-14

**Authors:** Wanchaloem Nadda, Waraporn Boonchieng, Ekkarat Boonchieng

**Affiliations:** 1 Department of Computer Science, Faculty of Science, Chang Mai University, Chiang Mai, 50200 Thailand; 2 Faculty of Public Health, Chiang Mai University, Chiang Mai, 50200 Thailand; 3 Center of Excellence in Community Health Informatics, Department of Computer Science, Faculty of Science, Chiang Mai University, Chiang Mai, 50200 Thailand

**Keywords:** Long short-term memory, Dengue detection, Influenza detection, Text mining

## Abstract

Symptom-based machine learning models for disease detection are a way to reduce the workload of doctors when they have too many patients. Currently, there are many research studies on machine learning or deep learning for disease detection or clinical departments classification, using text of patient’s symptoms and vital signs. In this study, we used the Long Short-term Memory (LSTM) with a fully connected neural network model for classification, where the LSTM model was used to receive the patient’s symptoms text as input data. The fully connected neural network was used to receive other input data from the patients, including body temperature, age, gender, and the month the patients received care in. In this research, a data preprocessing algorithm was improved by using keyword selection to reduce the complexity of input data for overfitting problem prevention. The results showed that the LSTM with fully connected neural network model performed better than the LSTM model. The keyword selection method also increases model performance.

## Introduction

Symptom-based machine learning models help patients self-detect diseases via electronic devices such as smart phones or robots in hospitals with automated question and answer systems [7]. Recently, several studies improved the text classification model for clinical department classification [27] and disease detection [12]. These studies used text from symptoms and other features of patients for disease detection [17].

Dengue fever (a mosquito-borne viral disease) [18] and influenza are dangerous infectious diseases that many people contract. Dengue and influenza have symptoms like the common cold, but they can be fatal. It is estimated that 3 to 5 million people each year become seriously ill due to influenza [21].

The research about machine learning or deep learning for dengue and influenza is divided into two parts, improvement prediction models for forecasting the number of patients [25] or forecasting an outbreak [8] in some areas or countries such as China [26], India [16], and Thailand [22]. Another type of research is focused on improving machine learning or deep learning models for detection of dengue fever and influenza from vital signs [6] and symptoms [1] of patients.

The Long Short-term Memory (LSTM) model is a recurrent neural network model. It is commonly used in text classification [13], time series classification [11], and time series forecasting [25].

In this research, we will use the LSTM model to classify the symptoms of patients as text. The LSTM model was concatenated with a fully connected neural network to use patient vital signs and other features as input data, including gender, body temperature, and age of patients to increase the performance of the classification model. Moreover, we improve our method for data preprocessing by removing words that are not important to classification, this simplifies the input data.

## Theorical foundations

In this section, we describe all of the methods we used for modeling in this research.

### Mutual information metric

Mutual information metric (MI) is a value used to show the ability to classify each keyword. We use MI to measure the correlation between each keyword and each class. Mutual information metric is denoted by MI(*w*, *c*), where *w* is a word and *c* is a class. It is calculated by Eq. (1).

1 $$ \mathrm{MI}\left(w,c\right)=\log \frac{f_A\bullet N}{\left({f}_A+{f}_C\right)\left({f}_A+{f}_B\right)} $$

When *f*_*A*_ is the number of documents in class *c* that contain word *w*, *f*_*B*_ is the number of the documents not in class *c* that contain word *w*, *f*_*C*_ is the number of the documents not in class *c* that do not contain word *w*. and *N* is the number of all documents. The MI(*w*, *c*) has a value in range [ − log(*N*), log(*N*)] this is shown in (2) and (3).

2 $$ \mathrm{MI}\left(w,c\right)=\log \frac{f_A\bullet N}{\left({f}_A+{f}_C\right)\left({f}_A+{f}_B\right)}\le \log \frac{N}{\left({f}_A+{f}_B\right)}\le \log (N) $$

3 $$ \mathrm{MI}\left(w,c\right)=\log \frac{f_A\bullet N}{\left({f}_A+{f}_C\right)\left({f}_A+{f}_B\right)}\ge \log \frac{f_A}{\left({f}_A+{f}_C\right)}\ge \log \frac{f_A}{\left({f}_A+{f}_C\right)}\ge \log \frac{1}{N}=-\log (N) $$

The MI of each word can be measured by finding the MI between the word and the class with the highest MI value. It is shown in Eq. (4) where *d* is the number of classes.

4 $$ \mathrm{MI}(w)=\underset{i=1:d}{\max}\mathrm{MI}\left(w,{c}_i\right) $$

The MI is the largest in the case of *f*_*A*_ = 1, *f*_*B*_ = 0, and *f*_*C*_ = 0 .The words that have a frequency of 1 are important for classification.

### Word embedding

Word embedding is the method for representing each word with a vector of a real number. Word2vec [15] is a method of word embedding, where neighbors’ vectors of each word represents words with similar meaning. We can set the dimension of the vectors for each word when we train the word2vec model. If we use a pre-train word2vec model, we can use the principal component analysis (PCA) to reduce the dimension of the vector of words to the dimension that we want.

### Interpolation

**Fig. 1:**
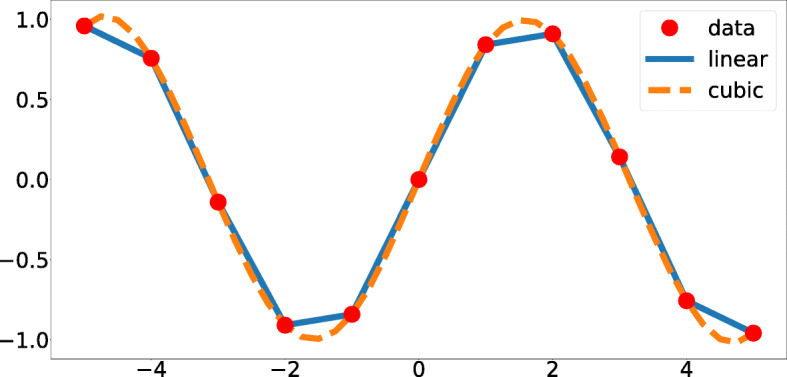
Data interpolation with linear and cubic functions

Interpolation is a method for estimating the missing data using polynomial or other functions [2], to obtain some points of data. An example for calculating the missing point of equation *y* =  *sin* (*x*) is shown in Fig. 1.

### LSTM

**Fig. 2:**
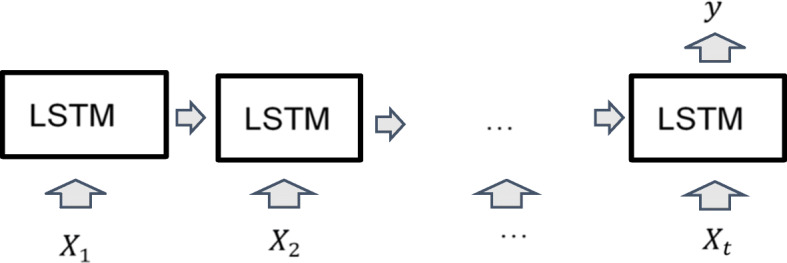
LSTM model structure

Long Short-term memory Neural Network (LSTM) [9] is a model architecture for recurrent neural network (RNN). The input data for each record of LSTM model is a sequence of vectors. A structure of LSTM is shown in Fig. 2 where *X*_*t*_ is a vector of input data with time stamp *t*.

The LSTM model is used for classification or prediction of sequential input data. In the present, the LSTM has had several improvements and has been used in several ways for time series prediction and text classification, such as LSTM fully convolutional networks for time series classification [11], bidirectional LSTM for sentiment analysis [13] and medical text classification [7].

### Imbalanced data problem

The imbalanced data problem is a problem of data classification, when the number of records in each class is vastly different [19]. In the case of binary class classification, we call the class with more records than the other class the majority class and call the other class the minority class.

There are two popular methods for solving the imbalanced data problem:

1. Using under sampling or oversampling for sampling training data in each class to have the same number of records.
2. Using some loss functions for machine learning or deep learning model to increase the weight of the minority class.

In this research we use the cost-entropy loss function [24] in Eq. (6) for the loss function of LSTM model for solving the imbalanced data problem. It has been improved upon from the cost-entropy loss in Eq. (5) where *t*_*k*_ = [*t*_*k*_(1), *t*_*k*_(2), …, *t*_*k*_(*d*) ] is the vector of target output of *k*^*th*^ record of dataset, *t*_*k*_(*i*) ∈ {0, 1} for *i* = 1, 2, …, *d*, and *y*_*k*_ = [*y*_*k*_(1), *y*_*k*_(2), …, *y*_*k*_(*d*) ] is the vector of output of model for *k*^*th*^ record of dataset, and *y*_*k*_(*i*) ∈ (0, 1) for *i* = 1, 2, …, *d*. Moreover, we set *n*_*k*_ to be the number of records of training data in the class of *k*^*th*^ record and set a constant value *γ* ∈ [0, 1].

5 $$ E=-\sum \limits_{i=1}^n\sum \limits_{k=1}^d{t}_k(i)\log {y}_k(i) $$

6 $$ E=-\sum \limits_{i=1}^n\sum \limits_{k=1}^d{t}_k(i)\log {y}_k(i){\left(\frac{1}{n_k}\right)}^{\gamma } $$

## Material and methods

### Data description

The data used in this research is from medical records from Saraphi Hospital, Chiang Mai Province, Thailand Between 2015 and 2020 [3–5]. We use only records of patients diagnosed with three diseases. This includes the common cold, flu, and dengue. We listed all the attributes we used in this research in Table 1.

**Table 1:** The attributes are used in this research

Attribute	Description
CHIEFCOMP	Text of symptoms of each patient
GENDER	Gender of each patient (0 = male, 1 = female)
MONTH_SERV	The month, that each patient comes to the hospital in each time.
BTEMP	Body temperature of each patient
AGE	Age of each patient (year of service minus by year of birth)

The distribution (average and standard deviation) of some features and the number of records for each class are shown in Table 2.

**Table 2:** The average, standard deviation, and number of patients for some features

Attributes\Classes		cold	Dengue	flu	all
AGE (years)	mean	36.188	27.269	32.6	36.002
	std	26.933	15.643	20.813	26.714
BTEMP (^°^*C*)	mean	36.793	37.248	37.784	36.824
	std	0.827	1.191	1.148	0.858
GENDER (records)	male	2188	25	64	2277
	female	2802	27	76	2905
number of records (records)		4990	52	140	5182
Length of sentence (words)	mean	7.930	5.096	5.771	7.843
	std	6.208	2.320	5.164	6.171
Number of words (words)		1279	102	158	1306
Word frequency (records)	mean	29.18	2.57	4.86	29.36
	std	159.19	3.32	10.58	161.66

From the statistical hypothesis test (t-test), it was found that:

1. Average of age: It was found that the mean of age of common cold patients was greater

than the mean of age of dengue and flu patients (*p*-value < 0.05), but the mean of age of dengue and flu patients was no different. (*p*-value > 0.05).

2) Average body temperature: It was found that the mean body temperature of common cold

patients were less than the mean of body temperature of dengue patients (*p*-value < 0.01), and the mean of body temperature of dengue patients was less than the mean of body temperature of flu patients (*p*-value < 0.01).

### Data preprocessing

In this research, the features used for classification include CHIEFCOMP, GENDER, MONTH_SERV, BTEMP, and AGE. For numerical features (BTEMP and AGE), we use min-max normalization to adjust the values in range [0,1]. Examples of data are shown in Table 3. For MONTH_SERV, we use one hot encoder to convert each value to a vector of integers. For the CHIEFCOMP column, the data in this column is a sentence in the Thai language. We use a python library “pythainlp” [20] for word tokenization. Here is an example of word tokenization, from the sentence “เป็นหวัดมีน้ำมูกไอ” (English: “Having a cold with a runny nose and cough”) to a list of words [“เป็น”, “หวัด”, “มี”, “น้ำมูก”, “ไอ”]. Then the python library “Gensim” [14] is used to create a word2vec model that converts the text of each record into a matrix of a real number.

**Table 3:** Examples of data in our dataset

Attribute	1st patient	2nd patient
CHIEFCOMP	เป็นหวัดมีน้ำมูกไอ (English: having a cold with a runny nose and cough)	ไข้ 6 วัน อ่อนเพลีย ไอเจ็บคอ (English: 6-day fever, weakness, cough, and sore throat)
GENDER	1	0
MONTH_SERV	08 (August)	03 (March)
BTEMP	36.5	38
AGE	50	23
DISEASE	cold	dengue

### Keywords selection

In the process of text preprocessing for LSTM training. We removed words that were not important for classification to simplify the incoming data including:

1. Low MI: words with low mutual information metric (bottoms 5%).
2. Low frequency: words with low frequency (frequency < 2) because it had high MI. That is, it has a high ability for classification. However, it may be a typographical error.

**Fig. 3:**
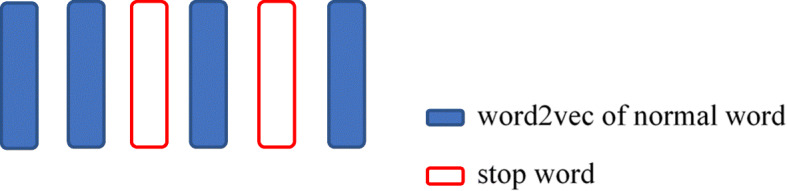
Vectors of words in a sentence after the removal of 2 stop words

These words are defined as stop words, and all stop words are removed from the data. Next, we set the positions of the removed words to missing values. It is shown in Fig. 3.

We use three methods to solve the missing values problem:

1. Cut the stop words: cut the vectors of all stop words in the sentence.
2. Fill with mean: fill the vectors of the missing values by the mean of word2vec of all words in the sentence with the corresponding position.
3. Interpolation: fill the vectors with the missing values by interpolation using the corresponding position in vectors.

**Fig. 4:**
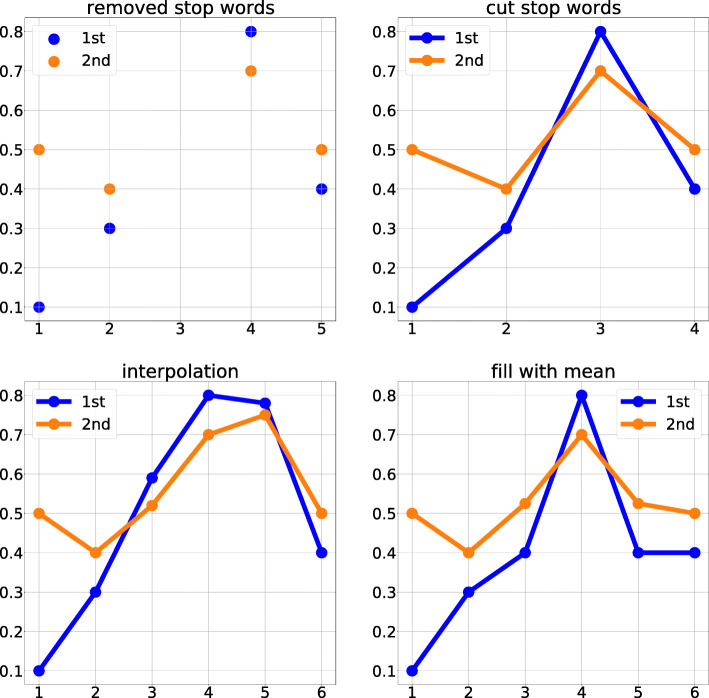
Solving missing values problem

We show the example of filling missing values for 2 dimensional word2vec vectors in Fig. 4.

### LSTM with fully connected neural network model

For training the models, we divide the data into 3 datasets including: training data, validation data, and testing data. At first, we use all of the words in the CHIEFCOMP column of the dataset to train the word2vec model, then we divide the dataset into two datasets: 80% training and validation data and 20% testing data.

**Fig. 5:**
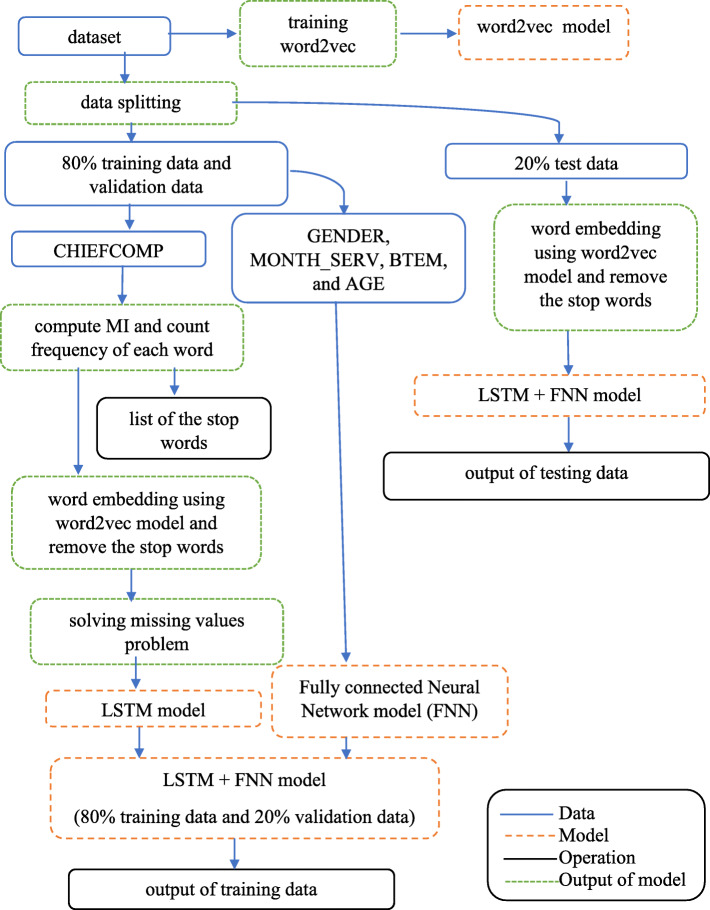
Research conceptual framework

In the next step, we find MI of all words in the training and validation dataset and then cut out the words that have low MI (bottom 5%) and cut out words with frequency less than 2. Next, we solve the missing values problem, and then use the training and validation dataset to train LSTM with the fully connected neural network model, by dividing the training and validation dataset into 80% training and 20% validation data. We show the conceptual framework for our research in Fig. 5. The softmax function in Eq. (7) is used as an activation function for the last layer of the classification model to compute probability of each record in each class where *y* = [*y*_1_, *y*_2_, …, *y*_*d*_ ] is a vector of real number.

7 $$ \mathrm{softmax}\left({y}_i\right)=\frac{y_i}{\sum \limits_{j=1}^d\exp \left({y}_j\right)} $$

## Results and discussion

### Performance measurement

Since the dataset in this research is an imbalanced dataset, we cannot use accuracy to measure the performance of the model. For this reason we use G-mean (geometric mean of recall) [28] for measurement of the performance models. G-mean is defined in Eq. (9) where d is the number of classes, recall(class *c*_*i*_) is a recall of class *c*_*i*_ defined in Eq. (8).

8 $$ \mathrm{recall}\left(\mathrm{class}\ {c}_i\right)=\frac{\mathrm{number}\ \mathrm{of}\ \mathrm{records}\ \mathrm{in}\ \mathrm{class}\ {c}_i\ \mathrm{that}\ \mathrm{true}\ \mathrm{class}\mathrm{ification}}{\mathrm{number}\ \mathrm{of}\ \mathrm{all}\ \mathrm{records}\ \mathrm{in}\ \mathrm{class}\ {c}_i\ } $$

9 $$ \mathrm{G}-\mathrm{mean}=\sqrt[d]{\prod \limits_{i=1}^d\mathrm{recall}\left(\mathrm{class}\ {c}_i\right)} $$

### Performance of model

We have shown the performance of all models in Tables 4 and 5. Label-indicator morpheme growth (MG) [10] is the method that adds weight to the keywords with the highest MI (top 5%). SMS spam dataset is the basic dataset for text classification [23]. The model used in this research, was single layer LSTM and single hidden layer neural network (5 hidden nodes) with Adam optimizer in python library “keras”.

**Table 4:** Performance of models -- Area under the ROC Curve (AUC)

	Dataset	dengue + cold			flu + dengue + cold			flu + cold			SMS Spam Collection Dataset
	Model Architecture / Filing missing	LSTM	LSTM with numerical	LSTM + FNN	LSTM	LSTM with numerical	LSTM + FNN	LSTM	LSTM with numerical	LSTM + FNN	LSTM
original		0.798	0.823	0.829	0.753	0.767	0.776	0.670	0.674	0.662	0.912
MG		0.797	0.808	0.826	0.750	0.757	0.779	0.678	0.670	0.676	0.913
keywords selection (cut words: frequency < 2)	cubic interpolation	0.798	0.791	0.817	0.728	0.755	0.738	0.734	0.770	0.795	0.920
	cut	0.803	0.831	0.837	0.723	0.778	0.782	0.671	0.675	0.658	0.922
	mean	0.802	0.800	0.825	0.719	0.781	0.760	0.729	0.743	0.692	0.959
keywords selection (cut words: MI bottom 5%)	cubic interpolation	0.744	0.628	0.684	0.622	0.675	0.594	0.691	0.808	0.831	0.930
	cut	0.712	0.641	0.754	0.689	0.753	0.716	0.653	0.616	0.551	0.929
	mean	0.721	0.637	0.718	0.681	0.769	0.717	0.686	0.776	0.673	0.968
keywords selection (cut words: MI bottom 5% or frequency < 2)	cubic interpolation	0.776	0.645	0.689	0.623	0.683	0.742	0.688	0.798	0.841	0.928
	cut	0.750	0.650	0.758	0.691	0.763	0.727	0.658	0.621	0.550	0.944
	mean	0.754	0.644	0.725	0.677	0.775	0.696	0.688	0.792	0.628	0.965
keywords selection (cut words: frequency < 2) + MG	cubic interpolation	0.794	0.784	0.809	0.743	0.752	0.742	0.665	0.755	0.818	0.916
	cut	0.794	0.807	0.845	0.764	0.759	0.786	0.682	0.670	0.675	0.904
	mean	0.801	0.784	0.826	0.769	0.778	0.771	0.669	0.713	0.717	0.952
keywords selection (cut words: MI bottom 5%) + MG	cubic interpolation	0.681	0.626	0.662	0.643	0.659	0.591	0.726	0.803	0.836	0.933
	cut	0.666	0.635	0.706	0.668	0.750	0.705	0.660	0.622	0.591	0.939
	mean	0.693	0.640	0.690	0.660	0.771	0.690	0.671	0.702	0.797	0.959
keywords selection (cut words: MI bottom 5% or frequency < 2) + MG	cubic interpolation	0.709	0.638	0.661	0.642	0.663	0.720	0.705	0.793	0.845	0.919
	cut	0.699	0.637	0.700	0.671	0.746	0.735	0.663	0.628	0.593	0.927
	mean	0.678	0.657	0.683	0.677	0.765	0.698	0.687	0.698	0.766	0.951

**Table 5:** Performance of models (G-mean)

	Dataset	dengue + cold			flu+ dengue + cold			flu+ cold			SMS Spam Collection Dataset
	Model Architecture / Filing missing	LSTM	LSTM with numerical	LSTM + FNN	LSTM	LSTM with numerical	-LSTM + FNN	LSTM	LSTM with numerical	LSTM + FNN	LSTM
original		0.692	0.800	0.771	0.458	0.586	0.543	0.528	0.532	0.565	0.854
MG		0.729	0.766	0.773	0.588	0.592	0.531	0.526	0.524	0.481	0.852
keywords selection (cut words: frequency < 2)	cubic interpolation	0.768	0.752	0.779	0.488	0.533	0.513	0.649	0.682	0.695	0.820
	cut	0.758	0.798	0.808	0.402	0.594	0.601	0.530	0.532	0.565	0.849
	mean	0.732	0.699	0.742	0.498	0.569	0.568	0.624	0.682	0.608	0.894
keywords selection (cut words: MI bottom 5%)	cubic interpolation	0.734	0.545	0.649	0.300	0.383	0.286	0.670	0.719	0.744	0.871
	cut	0.690	0.569	0.697	0.522	0.546	0.460	0.317	0.397	0.574	0.860
	mean	0.641	0.613	0.599	0.542	0.505	0.528	0.678	0.701	0.607	0.896
keywords selection (cut words: MI bottom 5% or frequency < 2)	cubic interpolation	0.715	0.628	0.599	0.372	0.392	0.439	0.667	0.687	0.757	0.851
	cut	0.668	0.568	0.714	0.497	0.556	0.478	0.319	0.357	0.569	0.886
	mean	0.714	0.599	0.605	0.488	0.555	0.560	0.676	0.675	0.609	0.900
keywords selection (cut words: frequency < 2) + MG	cubic interpolation	0.763	0.759	0.762	0.466	0.532	0.529	0.565	0.671	0.711	0.849
	cut	0.725	0.723	0.818	0.482	0.576	0.528	0.526	0.526	0.483	0.845
	mean	0.738	0.728	0.782	0.584	0.596	0.553	0.621	0.654	0.673	0.876
keywords selection (cut words: MI bottom 5%) + MG	cubic interpolation	0.698	0.606	0.620	0.222	0.000	0.282	0.661	0.728	0.754	0.864
	cut	0.680	0.655	0.641	0.484	0.555	0.455	0.402	0.358	0.495	0.876
	mean	0.697	0.621	0.590	0.490	0.501	0.477	0.656	0.642	0.766	0.894
keywords selection (cut words: MI bottom 5% or frequency < 2) + MG	cubic interpolation	0.703	0.604	0.581	0.293	0.000	0.405	0.677	0.722	0.716	0.845
	cut	0.671	0.609	0.642	0.483	0.537	0.504	0.364	0.359	0.492	0.836
	mean	0.697	0.621	0.590	0.478	0.563	0.529	0.678	0.647	0.666	0.884

For the LSTM model, we use LSTM with no hidden layer and LSTM with single hidden layer (the size of the vector in the hidden layer is 5) for performance comparison. In addition, for the single hidden layer fully connected neural network model, we ran the number of hidden nodes as 5, 10, 15, and 20. Moreover, for the word2vec model, we ran the size of the vector as 20, 25, and 30.

We considered our dataset in three ways, two of which are binary classes. It consists of 1) the common cold and dengue class, 2) the cold and flu class, and the other dataset is the multiple class (common cold, dengue and influenza class). For the SMS spam collection dataset, which is a standard dataset used to test the performance of our method. It consists of two classes, include ham and spam message.

**Fig. 6:**
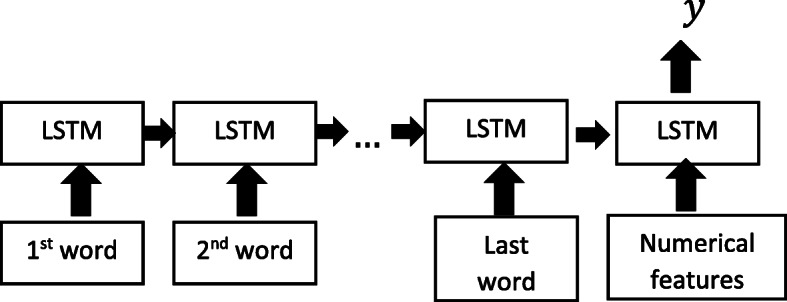
LSTM model structure

In addition to use the LSTM and LSTM with a fully connected neural network. We also used the LSTM model with numerical features as shown in Fig. 6. to compare the model’s performance.

The results showed that LSTM with a fully connected neural network had better performance than normal LSTM. Moreover, removing stop words increased the G-mean value of the testing data for all datasets. For the medical records dataset, LSTM with Fully connected Neural network gives the best G-mean value when words with low MI (bottom 5%) and low frequency (frequency < 2) together are considered stop words. If we set the stop words to be the words with low frequency (frequency < 2), then it reduces the training time (shown in Table 6) and increases the performance of the LSTM model. Moreover, LSTM with the feed forward fully connected neural network model uses less time for training than the LSTM model, because it has a faster convergence.

**Table 6:** The time of data preprocessing + models training and testing of each model (second). Run on data science server at Chiang Mai University, Thailand (LINUX VPS, RAM 16 GB, CPU INTEL CORE i9, GPU 2080TI 11GB)

	Dataset	dengue + cold			Flu + dengue + cold			Flu + cold			SMS Spam Collection Dataset
	Model Architecture / Filing missing	LSTM	LSTM with numerical	LSTM + FNN	LSTM	LSTM with numerical	LSTM + FNN	LSTM	LSTM with numerical	LSTM + FNN	LSTM
original		13.3	17.7	8.6	11.9	15.7	10.1	26.3	27.6	12.7	98.5
MG		11.6	17.1	8.6	10.7	14.0	10.1	26.2	27.8	18.4	85.5
keywords selection (cut words: frequency < 2)	cubic interpolation	12.4	12.3	12.9	55.2	47.5	10.9	24.0	24.7	20.6	114.2
	cut	13.4	17.6	8.6	10.9	17.1	11.7	26.3	27.6	12.5	107.7
	mean	14.3	16.7	8.5	11.6	17.8	12.0	23.6	16.5	8.0	101.5
keywords selection (cut words: MI bottom 5%)	cubic interpolation	22.5	14.5	12.3	48.9	29.3	10.9	24.1	25.2	21.5	171.0
	cut	22.9	15.6	11.5	12.5	19.3	10.2	24.9	25.7	12.8	111.0
	mean	25.2	12.2	10.9	16.8	22.8	10.8	19.9	20.0	8.5	116.6
keywords selection (cut words: MI bottom 5% or frequency < 2)	cubic interpolation	13.4	14.7	13.0	49.0	29.3	11.0	24.8	25.3	20.0	111.6
	cut	26.4	20.3	11.6	12.7	22.4	10.3	25.1	26.1	12.7	145.2
	mean	25.1	12.6	10.7	22.1	22.2	10.8	21.7	19.9	8.1	105.3
keywords selection (cut words: frequency < 2) + MG	cubic interpolation	11.2	12.2	12.2	55.3	47.2	11.0	22.3	16.5	22.1	134.7
	cut	11.5	16.9	8.7	10.6	13.8	10.2	26.6	27.6	18.1	87.5
	mean	14.7	14.3	8.5	14.2	19.2	12.5	24.1	14.3	8.0	116.7
keywords selection (cut words: MI bottom 5%) + MG	cubic interpolation	23.9	15.1	11.1	27.6	28.9	10.9	24.0	25.3	20.0	206.7
	cut	18.3	15.7	8.5	12.2	16.4	10.2	24.8	29.0	15.6	219.0
	mean	25.0	11.8	10.2	16.6	20.6	11.1	15.4	16.7	15.4	116.8
keywords selection (cut words: MI bottom 5% or frequency < 2) + MG	cubic interpolation	13.4	13.6	11.0	36.0	29.5	10.8	23.9	25.4	18.4	147.3
	cut	25.2	15.4	8.6	12.7	16.4	10.2	25.0	29.1	15.4	141.9
	mean	19.7	11.8	8.5	15.8	20.6	10.9	21.6	17.0	8.0	80.2

## Conclusion

This research used the LSTM model with fully connected neural network for dengue fever and influenza detection. Text of symptoms and other features including age, body temperature, gender, and month of service were used for input data. The results showed that the LSTM with the fully connected neural network model had higher performance than the normal LSTM model. In addition, removing unimportant keywords from the dataset and also increased their performance.

## Data Availability

Both programming code (python) and data are available upon request (ekkarat.boonchieng@cmu.ac.th).
